# Experimental Evaluation of a Universal Adhesive Modified With Fluorinated Graphene Nanoparticles: An In Vitro Study

**DOI:** 10.7759/cureus.113905

**Published:** 2026-08-03

**Authors:** Sadhika Pentapati, Ravichandra Ravi, Jyothi Mandava, Ravikumar Konagala, Sahithi Pamidimukkala, Sruthi Kapu

**Affiliations:** 1 Department of Conservative Dentistry and Endodontics, Gandhi Institute of Technology and Management (GITAM) Dental College and Hospital, Visakhapatnam, IND

**Keywords:** confocal laser scanning microscopy (clsm), fourier transform infrared spectroscopy (ftir), graphene nano fluoride particles, hybrid layer, resin tags, universal bonding agent

## Abstract

Background

Recent developments in nanotechnology have made it possible to add nanoparticles to dental materials, leading to improved physical and biological characteristics. The use of fluorinated graphene nanoparticles (FGNPs) has attracted attention due to their high surface area, chemical stability, and potential bioactive effects, which can improve adhesion and reduce problems related to bonding.

Aim

To evaluate and compare the chemical and interfacial effects of incorporating fluoride graphene nanoparticles into a universal adhesive system using Fourier-transform infrared spectroscopy (FTIR) and confocal laser scanning microscopy (CLSM) analysis.

Materials and methods

Thirty-two human extracted mandibular molars were randomized into two groups (16 for each group). The two groups were treated with 37% phosphoric acid on dentin and then a conventional universal adhesive or a modified universal adhesive with 2% FGNPs was applied. For FTIR and CLSM evaluation, the samples were further subdivided into groups (n = 8). Independent-sample t-test was used to analyze the data at a significance level of p<0.05.

Results

Analysis with FTIR showed characteristic C-F peaks with reduced O-H intensity in modified adhesive with FGNPs. The hybrid layer thickness and resin tag length was significantly greater in the modified adhesive group compared with the unmodified adhesive group with P=0.0001 and 0.0003 respectively.

Conclusion

The incorporation of FGNPs in universal adhesive has improved the chemical bonding and micromechanical interlocking, with enhanced hybrid layer thickness at dentin adhesive interface.

## Introduction

The long-term durability of the adhesive restoration mainly depends on the efficiency of the resin-bonding process to dentin. Despite the improvements, in modern dentin bonding agents, degradation of the hybrid layer continues to pose a significant problem [1]. The degradation process is mainly caused by water-induced breakdown of the bond, microleakage or enzymatic action of the matrix metalloproteinases (MMPs) found in dentin [2].

The universal adhesives were formulated to make bonding procedure easier while improving their versatility in different clinical situations. However, their longevity could still be affected by issues like the inadequate infiltration of resins, phase separation, and hybrid layer breakdown. The most vulnerable region for bonding would be the hybrid layer since the exposed collagen fibrils are subject to enzymatic dissolution [2].

In an effort to overcome these obstacles, scientists have started utilizing nanomaterials in adhesive technology. The small size of the particles helps strengthen materials, decrease their moisture absorbance, and improve their bonding performance [3]. One type of nanomaterial that has shown great promise is fluorinated graphene nanoparticles (FGNPs) [4,5].

FGNPs are two-dimensional, flat, fluorinated derivatives of graphene materials, which have hydrophobic properties but at the same time show high stability and mechanical resistance [2,3]. Due to the large surface area of FGNPs, there will be sufficient interactions with dentin and resin, leading to increased bond formation [2]. Moreover, FGNPs have the capacity to serve as an interface between hydrophilic dentin and hydrophobic resin, resulting in an increase in both chemical bonds and mechanical resistance.

Despite promising early studies, research is still lacking in the field of FGNP’s impact on bonding performance. Hence, this study aimed to evaluate and compare the chemical interactions of fluoride graphene nanoparticles incorporated into a universal adhesive system using Fourier-transform infrared spectroscopy (FTIR) and their influence on hybrid layer thickness and depth of resin tag formation using confocal laser scanning microscopy (CLSM) analysis. Thus, the null hypothesis tested was that the incorporation of FNGPs into a universal adhesive system would not produce any significant difference in hybrid layer thickness or resin tag depth formation compared with the conventional universal adhesive system.

## Materials and methods

Study design

The research protocol was approved by the Institutional Ethics Committee (IEC/GDCH/2026/SS/04-02). Thirty-two human mandibular molars that were extracted due to periodontal reasons were included in the study. Only teeth that were intact, free of decay, fully developed, and without any restorations or cracks were included. Teeth with cavities, fractures, or previous dental work were not used. The estimation of sample size was calculated using G*Power software (version 3.1; Heinrich Heine University Düsseldorf, Düsseldorf, Germany), based on data from an earlier similar study [1].

Preparation of fluoride graphene nanoparticles

Fluoride-doped graphene oxide (2% F-GO) was prepared using a wet impregnation technique. Firstly, 490 mg of graphene oxide was mixed into 20 mL of distilled water and sonicated for 40 minutes to create an even suspension. Meanwhile, 22.1 mg of sodium fluoride (NaF) was dissolved in 15 mL of water and slowly added to the graphene oxide mixture while keeping the temperature at 60°C and stirring continuously. This blend was kept stirring for three to four hours before being heated to 100°C to evaporate the liquid.

Preparation of modified universal adhesives

About 2w%, 30mg of fluoride-doped graphene nanoparticles (FGN) were introduced into 3 mL of universal adhesive solution (Tetric N-Bond Universal; Ivoclar, Schaan, Liechtenstein) [6]. Initially the solution was magnetically stirred for one minute in order to achieve an even dispersion of the FGNPs and kept in the ultrasonic bath for an hour in order to achieve a homogeneous mixture.

Sample preparation

The teeth were mounted in the acrylic block with dimensions of 1.5 cm depth, length and width to the level of the cement-enamel junction exposing the entire crown of the tooth. A flat surface for bonding procedure was obtained by cutting the occlusal surface of the crown about 1 mm below the dentin-enamel junction using low-speed cutting with finer metal abrasive disks. Each dentin surface was then polished with 600-grit silicon carbide paper under running water for 60 seconds to create a standardized smear layer.

Surface bonding procedure

All 32 teeth occlusal dentin surfaces were conditioned with 37% phosphoric acid gel (N-Etch; Ivoclar) for 15 seconds and rinsed with distilled water for 10 seconds to remove any residual etchant. The specimens were then randomly divided into two groups (n = 16 each). One group of samples received the unmodified universal adhesive, while the other received the modified universal adhesive with FGNPs. For CLSM evaluation of hybrid layer and resin tag formation, half of the specimens from each group (n = 8) were bonded using the respective modified or unmodified universal adhesive after incorporating 0.3 mg of rhodamine B fluorescent dye into 3 mL of the adhesive prior to application.

**Figure 1:**
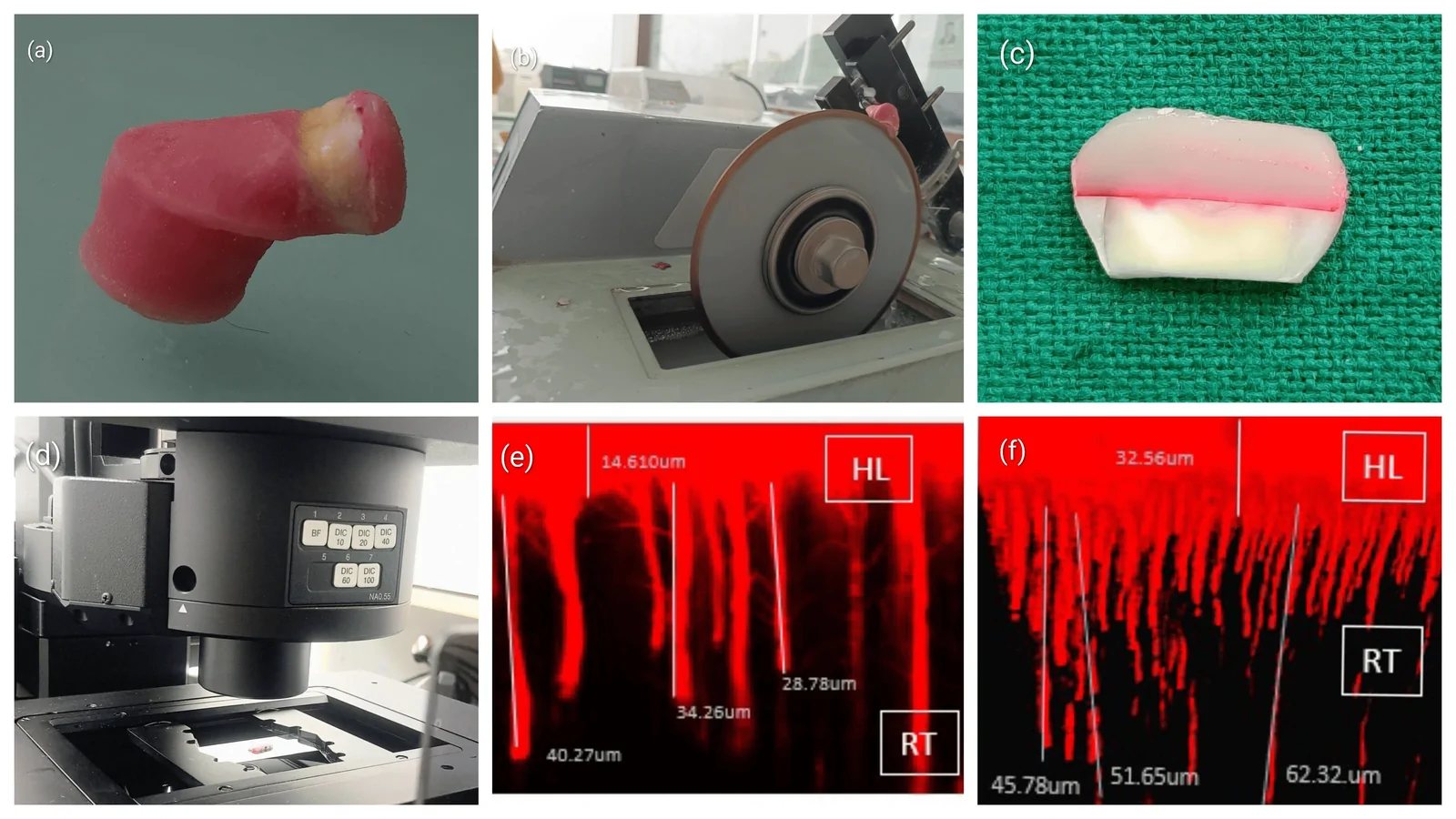
(a) Mounting of prepared sample (b) Microtome with tooth attached for sectioning (BAINCUT LSS, Model LSS003; Chennai Metco Pvt. Ltd., Chennai, India) (c) Tooth sectioned sample (d) Visualizing sample under confocal laser scanning microscope (Leica DMi8; Leica Microsystems GmbH, Wetzlar, Germany) (e and f) Confocal laser scanning microscope images of universal adhesive and modified universal adhesive with fluorinated graphene nanoparticles (FGNPs).

The adhesive was applied evenly using a microbrush, followed by gentle air thinning for 10 seconds to facilitate solvent evaporation. Polymerization was carried out for 20 seconds using an LED light-curing unit positioned approximately 5 mm from the dentin surface, with an output intensity of 600 mW/cm². Following adhesive placement and curing, a 4-mm composite resin build-up was constructed and light-cured for 20 seconds. The specimens were then prepared for microscopic assessment of hybrid layer formation and resin tag penetration (Figure 1).

CLSM evaluation

Specimens assigned for CLSM analysis were examined under a confocal laser scanning microscope at 40× magnification using a laser wavelength of 543-561 nm, corresponding to the excitation range of rhodamine B dye. Serial optical sections (Z-stack images) of the dentin-adhesive interface were captured to assess hybrid layer thickness and resin tag penetration depth (Figure 1). The data obtained was subjected to statistical analysis using the independent-samples t-test, with the level of significance set at p < 0.05.

## Results

Following bonding procedures, the specimens designated for FTIR analysis were positioned directly onto the attenuated total reflectance (ATR) diamond crystal of the FTIR spectrometer (Spectrum II, Bruker, Germany). The internal reflection element enabled assessment of the superficial region of the dentin-adhesive interface, facilitating the evaluation of chemical interactions occurring within the interfacial zone [7].

The FTIR results indicated that spectrum showed characteristic peaks corresponding to O-H stretching around 3400 cm⁻¹, C-H stretching near 2920 cm⁻¹, and C=C stretching around 1620 cm⁻¹, indicating the presence of the graphene framework.

**Figure 2:**
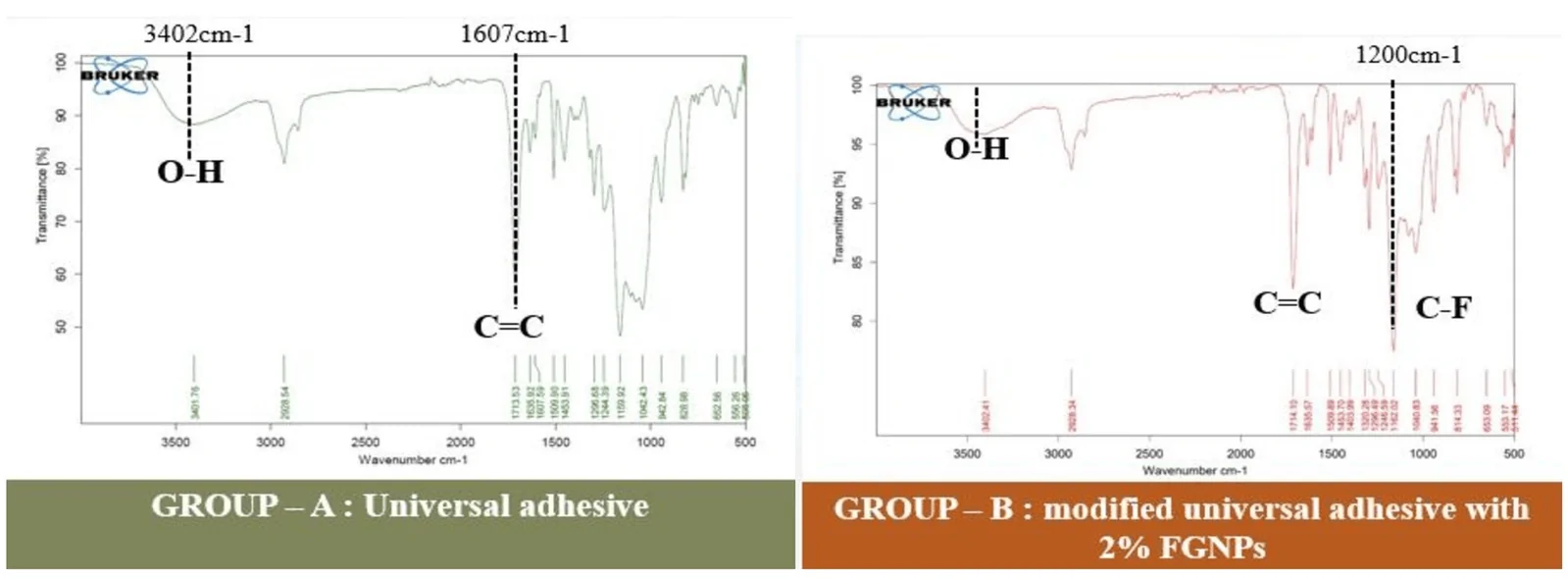
Group A: Fourier-transform infrared spectroscopy (FTIR) analysis of universal adhesive. Group B: FTIR analysis of modified adhesive with fluorinated graphene nanoparticles (FGNPs).

A distinct peak observed in the region of 1100-1200 cm⁻¹ corresponded to C-F stretching vibrations, confirming successful fluoride incorporation. These findings demonstrate the formation of stable fluoride graphene nanoparticles with appropriate functional group interactions (Figure 2) [5].

The CLSM results revealed that the average thickness of the hybrid layer formed with modified adhesive was significantly greater (P=0.0001) compared to unmodified universal adhesive (Table 1).

**Table 1:** Comparison of hybrid layer thickness scores in µm by independent t-test.

Group	n	Mean	SD	SE	t-value	P-value
Universal adhesive	8	13.50	0.76	0.27	22.1360	0.0001*
Adhesive with fluoride graphene nanoparticles	8	24.16	1.13	0.40		

A similar pattern was also seen in resin tag length which is formed with FGNP-modified adhesive and was significantly longer (P=0.0003) compared to unmodified universal adhesive, indicating improved penetration of the adhesive into dentinal tubules (Table 2).

**Table 2:** Comparison among the groups with resin tag length scores in µm by independent t-test.

Group	n	Mean	SD	SE	t-value	P-value
Universal adhesive	8	25.01	1.22	0.43	13.1116	0.0003*
Adhesive with fluoride graphene nanoparticles	8	31.78	0.80	0.28		

## Discussion

The incorporation of nanofillers into adhesive systems has been shown to reinforce the organic matrix of resin adhesives, thereby improving their mechanical properties [8]. A stronger hybrid layer reduces water sorption and limits hydrolytic degradation, ultimately decreasing the rate of bond deterioration over time. In the present study, the improved uniformity of the hybrid layer observed in the FGNP group supports this concept. A homogeneous interface ensures better stress distribution under functional loading, reducing the risk of interfacial failure [9-11].

According to the results obtained in this study, it can be determined that fluoride graphene nanoparticles and their addition to the universal adhesive significantly contributed to changes in the hybrid layer thickness, or resin tag depth formation compared to the conventional universal adhesive alone, therefore the null hypothesis was rejected. Fluoridation of graphene nanoparticles refers to the formation of fluoridated nanomaterials where fluorine atoms are bonded to the carbon layer in the form of stable C-F bonds. Such a chemical modification results in improved chemical stability, mechanical strength, and hydrophobic properties. As shown by FTIR spectra, fluoridated graphene nanoparticles successfully underwent integration into the matrix of the universal adhesive. The presence of strong peaks at wavelengths ranging from 1100 to 1200 cm⁻¹ is evident, indicating the existence of strong C-F bonds.

Moreover, it was noted that O-H bonds exhibited reduced signal intensities, which indicate reduced ability to attract water molecules. Furthermore, a reduction in O-H peak intensity was observed, indicating decreased water content at the interface. This finding is clinically significant, as residual moisture can interfere with polymerization, promote hydrolytic degradation, and accelerate bond failure. Reduced water uptake contributes to improved polymerization efficiency and long-term stability [3,4].

The thickness of the hybrid layer in the FGNP-modified adhesive group was relatively high compared to the unmodified adhesive. Thicker and well-defined hybrid layers signify better infiltration of adhesive components into the dentin matrix, indicating better adaptation of the material to the dentin interface [1]. This improvement is probably due to the smaller particle size and large surface area of the FGNPs, which facilitate their incorporation into the adhesive without affecting its viscosity.

Increased density and length of the resin tags in the modified adhesive group reveals that there was deeper infiltration of the resin into the dentinal tubules. The diffusion of FGNPs into the dentinal tubules and the dentin collagen network might have facilitated better micromechanical locking. The formation of resin tags increases the bonding surface area of the adhesive interface and might help improve its resistance to mechanical stresses. This finding has been observed previously with other types of graphene nanofillers [12,13].

The results obtained in this research are in accordance with previous findings by Maryoosh et al. [6], where adding 2% FGNPs to universal adhesives increased resin tag length leads to higher shear bond strength when the adhesives were used in an etch-and-rinse mode. Also, it was previously demonstrated that the addition of graphene to adhesives resulted in improved bonding and reduced leakage [3-5].

Another important mechanism underlying the enhanced performance of FGNP-modified adhesives is their ability to function as a molecular bridge. The polar C-F bonds and residual oxygen-containing functional groups on FGNP surfaces enable interaction with hydrophilic dentin components such as collagen and hydroxyapatite [14]. Simultaneously, the graphene interacts with hydrophobic resin monomers like bisphenol A-glycidyl methacrylate (Bis-GMA) and hydroxyethyl methacrylate (HEMA). This dual interaction strengthens the adhesive interface by improving chemical coupling between dentin and resin [15]. 

Apart from improving the mechanical characteristics of dentin adhesives, it is anticipated that FGNPs would provide significant antibacterial effects as additives. In a study by Bregnocchi et al. [16], it was found that dental adhesives containing graphene exhibited effectiveness in inhibiting the growth of Streptococcus mutans, thus reducing the chances of secondary caries. In another study by Sun et al. [14], it was observed that adding fluorinated graphene into restorative material improved antibacterial effects as well as mechanical properties. The antibacterial effect of FGNPs may be attributed to their capability to disrupt the bacterial cell membrane and hinder bacterial adhesion and biofilm formation [17].

Additionally, the gradual release of fluoride ions may help inhibit bacterial metabolism and acid production. These combined effects suggest that FGNP-incorporated adhesives may improve the longevity of restorations by protecting the adhesive-dentin interface from microbial colonization and recurrent caries [18]. The low wettability of the modified adhesive is expected to reduce moisture uptake and microbial colonization along the interface of the adhesive and dentin. These combined features would help improve the longevity of the restoration by minimizing microleakage and degradation of the bonded interface [12,18].

The limitation of the present study was that the current investigation took place in vitro under strictly controlled conditions, which cannot entirely emulate the biological oral environment, considering factors such as temperature changes, pH alterations, enzyme action, and cyclic loading.

## Conclusions

Under the constraints of the current study, the use of 2% FGNPs in dental adhesives was found to promote a thick hybrid layer and deeper resin tag formation, and improve the chemical bonding at the adhesive-tooth interface.
